# Past Actions as Self-Signals: How Acting in a Self-Interested Way Influences Environmental Decision Making

**DOI:** 10.1371/journal.pone.0158456

**Published:** 2016-07-22

**Authors:** Chang-Yuan Lee, Guy Hochman, Steven E. Prince, Dan Ariely

**Affiliations:** 1 Center for Advanced Hindsight, Duke University, Durham, North Carolina, United State of America; 2 School of Psychology, Interdisciplinary Center (IDC) Herzliya, Israel; University of Vienna, AUSTRIA

## Abstract

In the last few decades, awareness of environmental issues has increased significantly. Little has changed, however, in human activities contributing to environmental damage. Why is it so difficult for us to change our behavior in a domain that is clearly so important to the future of humanity? Here we propose and test the possibility that self-signaling, the way we view ourselves based on our past behaviors, is one of the factors contributing to the difficulty of taking environmental action. In three experiments, we show that previous self-interested thoughts or behaviors serve as important signals that hinder the likelihood of acting in line with an individual’s reported concern for the environment. This study not only helps explain the gap between environmental awareness and action, but also suggests alternative strategies for policymakers and environmental agencies to promote proenvironmental behavior.

## Introduction

Imagine that you find a cockroach on your kitchen floor. What would you do? Would you let it go, step on it or spray an entire can of pesticide on it? Household pesticides are the most common form of pest control in and around living areas, and 31.7% of American households use at least one form of pesticide a week [1,2]. Such widespread use raises serious concerns about the environmental downside of exposure to the chemicals contained in household pesticides [3].

According to instructions included on most home pesticide products, contact with the chemicals therein can cause symptoms ranging from dizziness to headache and nausea. On top of these mild immediate effects, research also shows that consistent exposure to household pesticides may have severe future consequences such as increased risk of cancer and childhood diseases [4–6]. For example, children who grow up in environments where higher levels of chemicals in household pesticides have accumulated (e.g., floor and yard areas) are at greater risk of neuroblastoma [4]. Moreover, the effect of pesticides may be related to longer durations of exposure in older children [4].

In the current paper, we examine the effect of past behavior on environmental decision-making and the willingness to engage in proenvironmental behavior. To that end, we examine a decision to use pesticides at home, when people are specifically told that these pesticides have hazardous consequences to the environment. Previous research has shown that most individuals have deep concerns about the environment [7–9]. For example, an international survey among 16 countries [7] found that both in developing and non-developing countries, the general attitude of the public is pro-environmental, and that people are willing to act in favor of the environment (see also [10]). Moreover, such proenvironmental tendencies increase when the harmful potential of environmental risks is highlighted [11–13]. For example, a field experiment found that consumers are less likely to use pesticides when environmental hazard and precaution information is added to the product [13]. Thus, it could be argued that individuals take information about environmental risks into account when making environmental decisions.

Despite this strong environmental concern, the decision to act in a proenvironmental manner (e.g., not using a hazardous pesticide) can become quite complex because preferences can be often ill-defined [14–16]. An ill-defined preference is the notion that people are unable to precisely evaluate the attractiveness of each alternative. For example, most people have no idea which brand of wine they prefer more than the others, so they tend to look at the prices of wines on the menu, make a relative judgment, and often end up picking the second cheapest wine on the list [17]. In the current study, we argue that self-signaling [18–22] plays an important role in such complex situations where decision-makers weigh their (ambiguous and ill-defined) environmental attitudes against personal concerns that are not easily and directly comparable [23].

The basic idea of self-signaling is that people see their previous actions as signals of who they are and what they care about [24,25]. Specifically, people infer their own preferences from their own past behavior. Bem‘s seminal work [19,18] is one of the first illustrations of this phenomenon. According to Bem, people often learn about their own preferences as they learn about others’ preferences, that is, through observations of their own behavior. Individuals examine their actions (e.g., “I eat brown bread”) and based on their behavior form their preferences (e.g., “I must like brown bread”). In this process of self-signaling, previous actions serve as guides for subsequent decisions. In line with this notion, Gino, Norton and Ariely [26] also showed that participants who believed they were wearing counterfeit sunglasses cheated more in a perceptual task relative to participants who believed they wore brand-name sunglasses. Wearing counterfeit sunglasses was used as a signal that one is not honest and as a consequence, participants behaved in a less honest way.

Further support for self-signaling could be found in cognitive consistency theories such as parallel constraint satisfaction [27] and the coherence effect [28,29]. For example, Simon et al. [29] show that preferences are constructed or changed to cohere with the decision at hand. These findings suggest that under complex situations, prior actions increase the impact of attributes favoring the consistent outcome and weaken the significance of attributes that favor alternative options, in order to increase internal coherence.

In the current study, we use the theoretical idea of self-signaling to examine how previous self-interested behavior (i.e., spraying hazardous pesticides) affects subsequent environmental decision-making. Self-signaling suggests that since people tend to use household pesticides from time to time, these previous actions might affect their willingness to act in a proenvironmental manner and increase their tendency to use hazardous pesticides. Moreover, people may use thoughts as well as actions as signals for defining who they are [30,31] and guiding future behaviors, even if these thoughts do not align with actual behavior [32,33]. For example, Kruger and Gilovich [32] showed that thinking about offering to help a friend with their homework makes people feel good about themselves, even if they didn’t go on to help their friend. Based on these findings, self-signaling may affect consequent behavior when people only consider certain courses of actions, even if they do not actually execute them.

Based on the rationale we developed above, we predict that proenvironmental concerns [8,7] can be overridden if people have taken a self-interested action (i.e. spraying household pesticides) in the past. In addition, we predict that simply considering taking a self-interested action (without actually executing it) can also signal individuals about their concern for the environment and hence influence their subsequent environmental decisions.

## Experiment 1

Experiment 1 had two aims. First, we tested our assumptions that participants would be less likely to use pesticides when the risk of pesticide usage to the environment is salient. We featured the risks by showing participants a hazard note attached to the description of the pesticide. Second, we examined whether engaging in a self-serving action that yields an immediate benefit reduces subsequent environmental concerns. Specifically, we measured whether people would be more likely to use the hazardous pesticide if they have already used it.

### Method

#### Ethics Statement

The study and the consent procedure were approved by Duke University’s Institutional Review Board. In accordance with the ethics protocol, participants provided electronic consent for participation at the beginning of the study.

#### Participants

134 U.S. participants (mean age = 30.8 years; 41% female) were recruited via Amazon Mechanical Turk in exchange for $0.10 compensation. Participants were randomly assigned to one of three conditions: the *no hazard note* condition, the *hazard note* condition and the *hazard note with prior action* condition.

#### Design and Procedure

Participants were presented with a web-based questionnaire, which included two stages that were administered under three between-subjects conditions.

In the first stage, participants were presented with a description of a new pesticide product called ‘Radar^®^ Bug Killer’. To highlight the delayed consequences of the pesticide, participants in the *hazard note* condition and the *hazard note with prior action* condition read the following note added to the description:

"Note: The chemical compounds are toxic to pets and plants and can cause environmental hazards."

After reading the description of the pesticide, participants in the *no hazard note* and the *hazard note* conditions immediately started the second stage.

Before moving on to the second stage, participants in the *hazard note with prior spraying* condition were asked to imagine that they had previously used Radar^®^ Bug Killer to kill a moth that morning. To mimic an actual action, participants in this condition were asked to click a red dot on the screen to simulate a spraying action. After completing this step, participants in the *hazard note with prior spraying* condition started the second stage of the experiment.

In the second stage, participants were asked to use a 7-point scale (1 = *very unlikely*, 7 = *very likely*) to indicate how likely they were to use the pesticide if they found a spider in their home (“Please imagine that you see a spider at your place now, how likely are you to use the spray?”).

In addition to the main experiment, a separate pretest (n = 62) was conducted to verify whether the addition of the hazard note to the pesticide description indeed increased concerns for environmental risks of using Radar^®^ Bug Killer. Participants were recruited from Amazon Mechanical Turk. They were asked to read the same description of pesticide with the *hazard note* (n = 31) or without the *hazard note* (n = 31) and to rate the perceived risk level to the environment of pesticide usage (“*How risky do you think Radar*^*®*^ *Bug Killer will be to the environment*?*”*(1 = not at all; 7 = very risky)).

### Results and Discussion

Manipulation check. The averaged perceived risk to the environment of pesticide usage was higher when the hazard note was present versus absent (*M* = 5.19, *SE* = 0.27 vs. *M* = 4.35, *SE* = 0.29). Independent-samples t-test revealed that this difference is significant (*t*(60) = 2.08, *p* < 0.05, *d*_*s*_ = 0.53). This result demonstrates that the hazard note condition increased the perceived environmental risks associated with the use of household pesticide.

**Fig 1:**
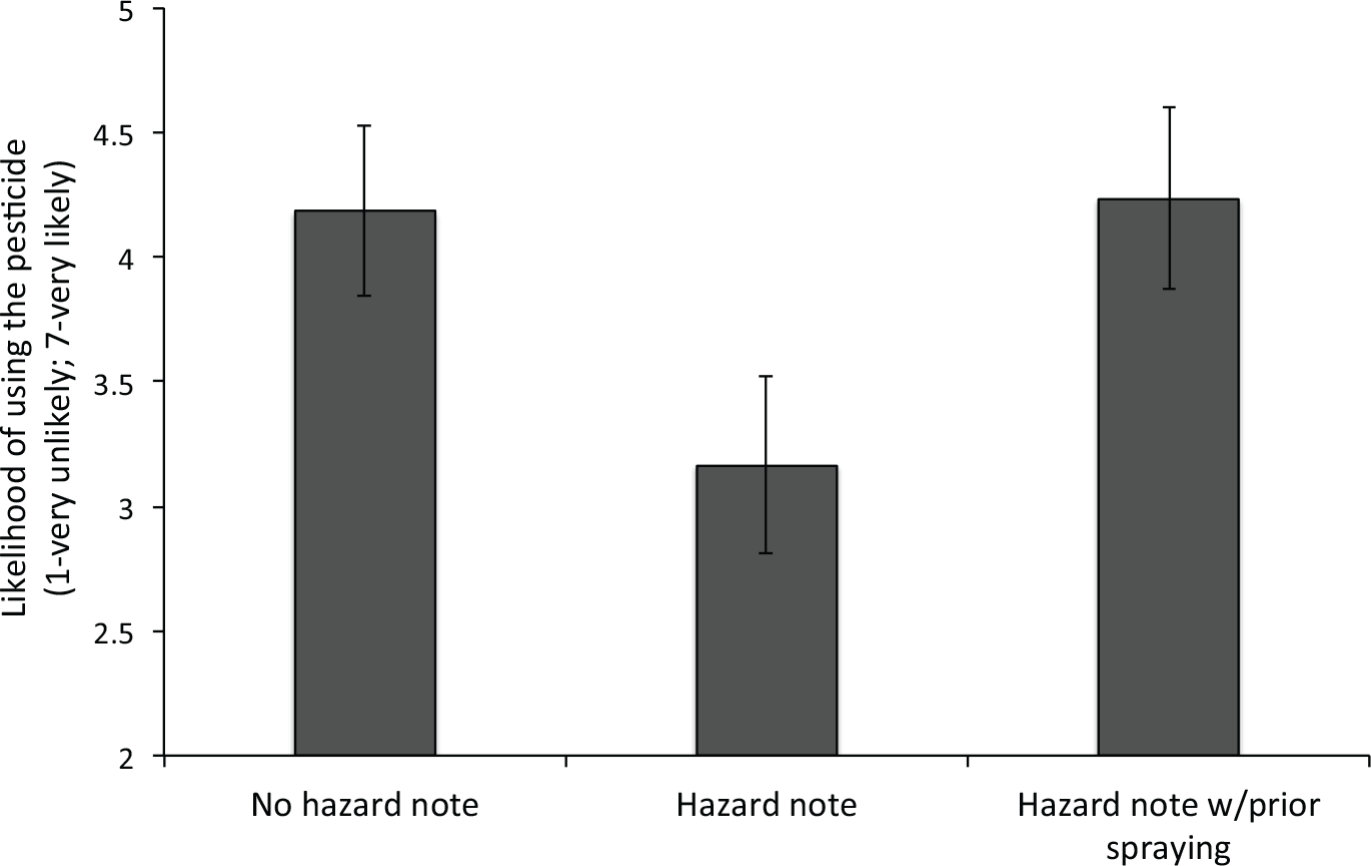
Averaged likelihood of using the pesticide as a function of the experimental condition in Experiment 1. Vertical lines represent standard errors.

Main results and discussion. The results of Experiment 1 are presented in Fig 1. The averaged likelihood of using *Radar*^*®*^ *Bug Killer* was 4.18 (*SE* = 0.34) in the *no hazard note* condition, and 3.17 (*SE* = 0.36) in the *hazard note* condition. The averaged likelihood of using the pesticide in the *hazard note with prior spraying* condition was 4.23 (*SE* = 0.36). One-way analysis of variance (ANOVA) revealed that the effect of the experimental condition was significant (*F*(2,131) = 2.80, *p* < 0.05, one-tailed; *η*^2^_*p*_ = 0.04). Planned contrasts further revealed that the likelihood of using the pesticide was significantly greater in the *no hazard note* condition and the *hazard note with prior spraying* condition than the *hazard note* condition (both *p*'s < 0.05; both *d*_*s*_'s > 0.40).

In line with previous findings, the tendency to use pesticides was significantly reduced when the risks to the environment were highlighted [13]. This pattern of results indicates stronger preferences for environmental considerations over personal benefits. However, when participants were asked to imagine that they used the hazardous pesticide earlier, an increased likelihood of using the pesticide was observed. In line with the self-signaling approach, this prior self-interested action might have signaled to participants that they care more about getting rid of pests than the negative environmental consequences their actions might cause.

## Experiment 2

In Experiment 1, we showed that a self-interested prior action might lead to a lasting and negative environmental decision-making. However, an alternative explanation for the increased likelihood of using the pesticide could be that initially killing bugs desensitized participants’ attitudes towards repeating such actions [34]. As a result, individuals may have been more likely to use the pesticide because they were less sensitized toward killing bugs in general. Experiment 2 was designed to juxtapose this alternative explanation (i.e., desensitization) with our self-signaling account. Specifically, we wanted to see if a prior action of killing a bug in a way that does not jeopardize the environment (e.g., stepping on a bug with one’s shoes) will also influence individuals' willingness to use environmentally hazardous pesticides, or if the effect is unique to previous pesticide usage.

### Method

#### Participants

Three hundred and thirty one U.S. participants (mean age = 34.14 years; 49% female) were recruited via Amazon Mechanical Turk in exchange for $0.10 compensation. Participants were randomly assigned to one of the six (between-subjects) experimental conditions.

### Design and Procedure

Participants were randomly assigned to one of six conditions in a 2 (hazard note: *yes* vs. *no*) × 3 (prior: *no prior* vs. *prior killing* vs. *prior spraying*) between-subjects design. As in Experiment 1, participants first read the pesticide description with or without the hazard note (depending on the hazard note condition), and with or without imagining that they had killed a moth earlier that morning (depending on the prior condition). As the main dependent variable, participants were asked to decide whether to use the “Radar^®^ Bug Killer” pesticide on a spider (“Please imagine that you see a spider at your place, how likely are you to use the spray?” 1 = *very unlikely*, 7 = *very likely*)

To test the desensitizing account, we added a *prior killing* condition, in which participants were asked to imagine that they had previously killed a moth by crushing it that morning with a paper towel. As in the *prior spraying* condition, participants in the *prior killing* condition were also asked to click a red dot on the screen (same as in the *prior spraying* condition) to simulate a crushing action (a spraying action in the *prior spraying* condition).

## Results and Discussion

A 2 × 3 ANOVA was first conducted to test the effect of the hazard note and the prior condition on the likelihood of using the pesticide. The analysis revealed a significant main effect for the hazard note (*F*(1,325) = 8.08, *p* < 0.01; *η*^2^_*p*_ = 0.02), and for the prior (*F*(2,325) = 5.32, *p* < 0.01; *η*^2^_*p*_ = 0.03). In addition, a significant interaction between these two factors was observed (*F*(2,325) = 4.21, *p* < 0.05; *η*^2^_*p*_ = 0.03).

**Fig 2:**
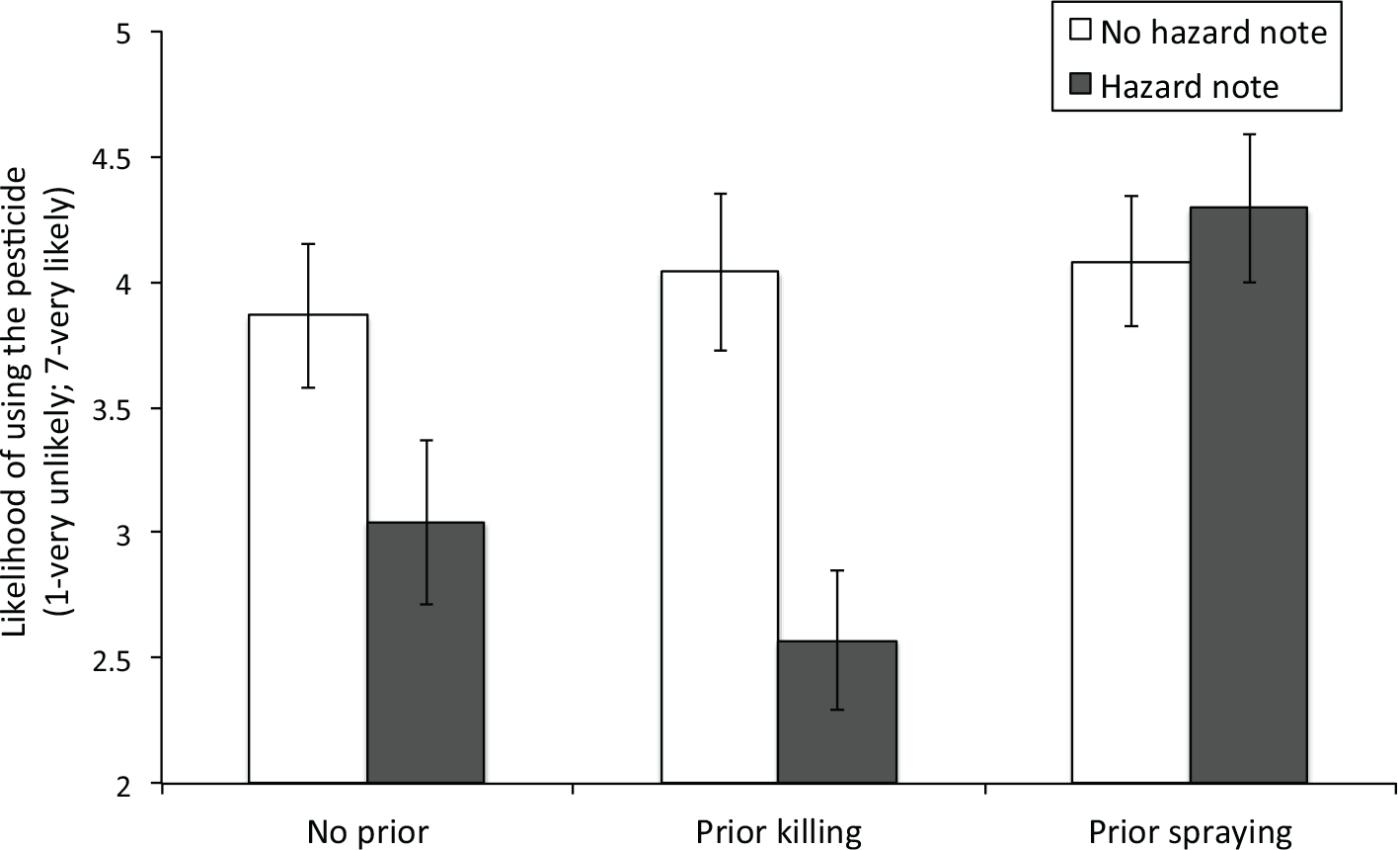
Averaged likelihood of using the pesticide as a function of the experimental condition in Experiment 2. Vertical lines represent standard errors.

As can be seen in Fig 2, the averaged likelihood of using Radar^®^ Bug Killer was lower for participants who read the hazard note than for those who did not read the note, both in the *no prior* (*M*_*hazard note*_ = 3.04, *SE* = 0.33; *M*_*no hazard note*_ = 3.86, *SE* = 0.29; *t*(103) = -1.89, *p* = 0.06, *d*_*s*_ = 0.37) and the *prior killing* conditions (*M*_*hazard note*_ = 2.57, *SE* = 0.28; *M*_*no hazard note*_ = 4.04, *SE* = 0.31; *t*(98) = -3.53, *p* < 0.01, *d*_*s*_ = 0.71). The effect of hazard note was marginally significant in the no prior condition and significant in the prior killing condition. As in Experiment 1, this pattern suggests that people are less willing to use a pesticide if it is harmful to their environment. By contrast, in line with our previous results, no effect of hazard note was found in the *prior spraying* condition (*M*_*hazard note*_ = 4.29, *SE* = 0.30; *M*_*no hazard note*_ = 4.08, *SE* = 0.28; *t*(124) = 0.52, *p* = 0.61, *d*_*s*_ = 0.09). Planned contrasts further revealed that when participants read the hazard note, the likelihood of using the pesticide in the *no prior* and the *prior killing* conditions was significantly lower than the *prior spraying* condition (vs. no prior: *t*(109) = -2.78, *p* < 0.001, *d*_*s*_ = 0.54; vs. prior killing: *t*(114) = -4.17, *p* < 0.001, *d*_*s*_ = 0.79). However, no difference was found between conditions when participants did not read the hazard note (*F*(2,166) = 0.16, *p* = 0.85, *η*^2^_*p*_ < 0.01).

This pattern of results suggests that killing bugs does not desensitize people and turn them into enthusiastic 'bug killers'. The results also show that self-interested concerns to eliminate the aversive bug did not outweigh concerns for the environment when participants imagined previously killing a bug. And most importantly, these results further support the finding that prior actions served as self-signals and affected environmental decision-making. In Experiment 3, we further examine this interpretation and test whether it holds when participants are only required to contemplate, but not execute, an action.

## Experiment 3

Experiment 1 and Experiment 2 show that a prior action with environmental consequences affects environmental decision-making. In Experiment 3, we examined if a mere thought of such behavior is enough to elicit this effect. Specifically, the aim of this experiment was to test if the self-signaling effect can be generalized to situations in which people only consider self-interested actions without actually acting on them.

### Method

#### Participants

One hundred and sixty U.S. participants (mean age = 34.62 years; 46% female) were recruited via Amazon Mechanical Turk completed a web-based study in exchange for $0.10 compensation.

### Design and Procedure

**Fig 3:**
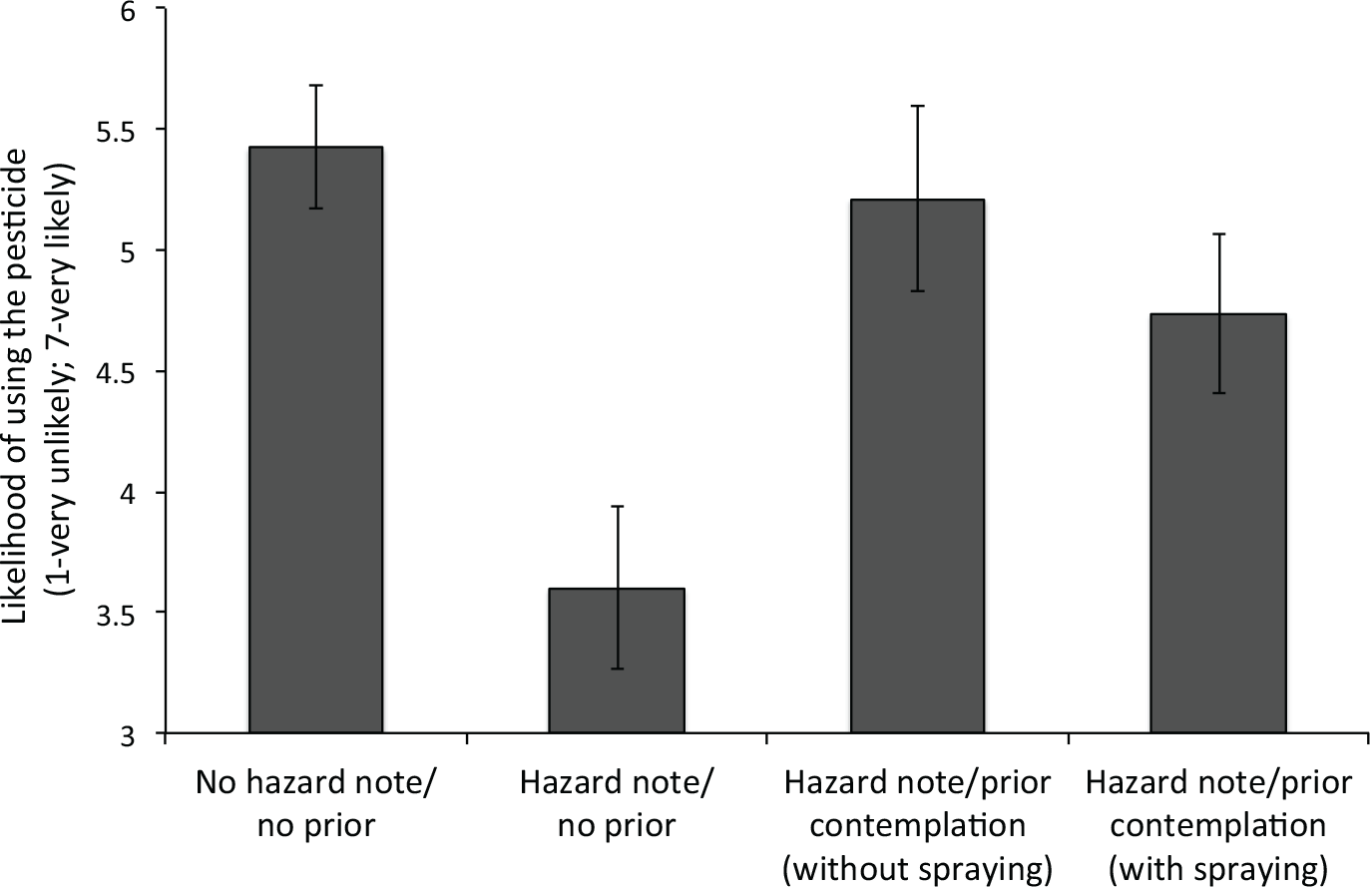
Averaged likelihood of using the pesticide as a function of the experimental condition in Experiment 3. Vertical lines represent standard errors.

Participants were presented with a two-stage questionnaire. In the first stage, participants were presented with the same pesticide description and hazard warning (if in the *hazard note* condition) as in Experiment 1 and Experiment 2. Participants were randomly assigned to one of four between-subjects conditions (see Fig 3).

After reading the description of the pesticide, participants in two *no prior* conditions immediately started the second stage. Participants in the two *prior contemplation* conditions were first asked to imagine that they noticed a moth earlier that day and thought about using the pesticide to kill it. Next, participants were asked to contemplate for a few seconds (*M* = 5.91 seconds, *SE* = 0.46) whether to use the pesticide to spray the moth. After indicating that they finished contemplating, participants were randomly assigned to one of two conditions. In the *prior contemplation with spraying* condition, participants were told that after contemplating, they have decided to use the pesticide. In the *prior contemplation without spraying* condition, they were told that they have decided not to use it. Thus, while the former condition is similar to the prior action condition in Experiment 1 and 2, the latter involves no action at all.

In the second stage, all participants were asked to rate how likely they were to use the pesticide on a cockroach (on a 7-point scale; 1 = *very unlikely*, 7 = *very likely*).

### Results and Discussion

The results of Experiment 3 are presented in Fig 3. As in Experiment 1 and 2, the reported likelihood of using the pesticide to eliminate an aversive bug was the highest in the *no hazard note/no prior* condition (*M* = 5.43, *SE* = 0.25), and lowest in the *hazard note/no prior* condition (*M* = 3.60, *SE* = 0.34). Moreover, as in the previous two experiments, the likelihood of using the hazardous pesticide increased in the *prior contemplation with spraying* condition (*M* = 4.74, *SE* = 0.33), supporting our self-signaling account. Importantly, the likelihood of using the pesticide also increased for the *prior contemplation without spraying* condition (*M* = 5.21, *SE* = 0.38). This pattern of results suggests that contemplating a self-interested action (even without acting upon it) was enough to affect this type of environmental decision-making.

A one-way ANOVA revealed that the effect of the experimental condition was significant (*F*(3,156) = 6.15, *p* < 0.01, *η*^2^_*p*_ = 0.11). In addition, planned contrasts further revealed a significant difference between the *no hazard note/no prior* and *hazard note/no prior* conditions (*t*(78) = -4.33, *p* < 0.001, *d*_*s*_ = 0.97), and the *prior contemplation with spraying* or the *prior contemplation without spraying* and the *hazard note/no prior* condition (*t*(80) = -2.41, *p* < 0.05, *d*_*s*_ = 0.53 and *t*(76) = -3.17, *p* < 0.01, *d*_*s*_ = 0.72, respectively). However, no difference was found between the *prior contemplation with spraying* or *without spraying* and the no *hazard note/no prior* condition (*t*(80) = -1.64, *p* = 0.11, *d*_*s*_ = 0.36 and *t*(76) = -0.64, *p* = 0.64, *d*_*s*_ = 0.11, respectively). Finally, the likelihood of using the pesticide was not different between the *prior contemplation with spraying* and the *prior contemplation without spraying* conditions (*t*(78) = -0.94, *p* = 0.35, *d*_*s*_ = 0.21), suggesting that previous self-interested thoughts have a similar effect as prior actions.

## General Discussion

The theory of self-signaling suggests that individuals learn about their preferences from their previous behavior [20–22], even if this behavior was driven by situational factors rather than personal preferences and beliefs [19,18]. As a result, these learned and inferred preferences from past behaviors can influence subsequent decisions.

In the current paper, we used the willingness to use household pesticides to examine whether past behavior can influence individuals’ concerns for the environment and give priority to immediate benefits. Specifically, we argued that past self-interested actions (e.g., using hazardous pesticide) serve as self-signals, and increase the impact of attributes that favor the self-interested outcome while decreasing the impact of attributes that favor environmental consequences.

Experiment 1 showed that, while people are concerned about the environment, a prior action yielding personal benefits (eliminating the aversive bug) leads them to prioritize self-interested actions at the expense of environmental protection. Experiment 2 further demonstrated that this pattern of results is not likely to be driven by mere desensitization to killing bugs. An increased preference to use the hazardous pesticide was only observed after a use of the hazardous pesticide but not after killing a bug with a paper towel. Finally, Experiment 3 suggested that merely considering a self-interested action might also signal individuals’ preferences.

Still, it could be argued that the current results stem from dissonance reduction. According to cognitive dissonance theory [35], a conflict between individuals’ preferences and behavior creates a psychological tension (dissonance). To resolve this tension, people might change their behavior, or change their preferences. According to cognitive dissonance, preferences might change to follow a certain behavior or behavior might change to adhere to initial preferences. In the current study, however, most participants were primarily motivated to change their actions based on their previous behavior, and not based on their environmental preferences (which were demonstrated by the reduced likelihood of using a hazardous pesticide). Moreover, the results were replicated in Experiment 3, where no action was made.

Our findings may help explain why, despite an increase in caring for the environment [8,7,9], the degree of pollution and overdevelopment remains high. Occasionally, people consider an action, or even take an action (e.g., using a pesticide to eliminate aversive bugs), that might not be good for the environment. As our data show, this self-interested behavior, which might signal that they give priority to the immediate benefits associated with the action over their concerns for the environment, can decrease the likelihood of future related proenvironmental behaviors.

Since our findings suggest that previous actions have the potential to influence future behavior, it seems that from an environmental protection perspective, a focus on raising awareness about environmental issues is not enough to make people act for the environment. We suggest that policymakers and proenvironmental agencies should encourage people to prevent a (first) lapse and to use sustainable products, which should signal to people that they care about future consequences for their environment.

Moreover, the theory of self-signaling suggests that encouraging people to recall their previous proenvironmental behavior or intention (as opposed to retrieving signals from their past hazardous actions that could engender environmental damage) may accordingly self-signal environmental concerns and subsequently motivate proenvironmental actions. For example, some credit card companies have a “Member since” date printed on their cards to highlight the seniority of their customers. In a similar vein, municipalities could harness the power of self-signaling to promote proenvironmental behavior. By making such behavior (e.g., recycling) more easy and accessible, they increase the likelihood that people will engage in proenvironmental behavior, and by highlighting these behaviors and making them more salient (e.g., issuing “Recycler since” cards or bumper stickers to their recycling citizens), they can ensure that these behaviors will persist.

The idea of self-signaling has much broader implications that should be tested in future research. For example, imagine a restaurant patron who is offered a free side dish of steamed broccoli or fresh salad with any lunch order. Similarly, consider schools that offer their students a complimentary healthy food at the beginning of the lunch line. In both cases, such an offer can signal to people that they care about their health and affect their future food decisions. As another example, if every child had a college savings account offered to them, it could signal that they value higher education and hence might increase the likelihood of attending college. Our results suggest that such facilitative interventions may encourage health, savings and other beneficial behaviors.

Finally, the idea of self-signaling might be relevant to the domain of moral decision-making. For example, pro-environmental behaviors are often considered an indication of moral behavior because they represent an intrinsic desire to do the right thing in order to benefit the environment [36–38]. On top of that, violations of moral obligations toward the environment can create a tendency to engage in immoral behavior due to a so called ‘what-the-hell-effect’ [26,39]. According to this effect, reduced sensitivity following immoral acts along with ego depletion [40,41] may lead people to be less moral. Our results indicate that this tendency may result from previous behavior that signals to someone that they are less moral than they thought, and thus exhibit less moral behavior.

## Supporting Information

S1 File The description of pesticide in all experiments. (PDF)

S1 Data Worksheet contains all the relevant data collected in Experiment 1. (CSV)

S2 Data Worksheet contains all the relevant data collected in Experiment 2. (CSV)

S3 Data Worksheet contains all the relevant data collected in Experiment 3. (CSV)
