# Supplementary material for: Past Actions as Self-Signals: How Acting in a Self-Interested Way Influences Environmental Decision Making
Source: PLoS One. 2016 Jul 22;11(7):e0158456. doi: 10.1371/journal.pone.0158456 (PMC4957805; doi:10.1371/journal.pone.0158456)
Supplement: S1 File — (PDF) [file pone.0158456.s001.pdf]

Radar® Bug Killer is specially formulated to kill crawling, flying and garden insects. Use it anywhere indoors or outdoors and around houseplants, and garden plants.

Instructions:

- *Shake well before using.*
- *Hold container upright and spray away from face.*
- *Do not enter or allow adults, children, or pets to enter the treated area until sprays have dried.*
